# A case of CD74‐ROS1‐positive lung adenocarcinoma diagnosed by next‐generation sequencing achieved long‐term survival with pemetrexed regimens

**DOI:** 10.1111/1759-7714.15041

**Published:** 2023-07-24

**Authors:** Satoshi Tanaka, Nobuaki Yoshimura, Ryo Asakawa, Satoshi Tobita, Moto Yaga, Kiyonobu Ueno

**Affiliations:** ^1^ Department of Respiratory Medicine Osaka General Medical Center Osaka Japan

**Keywords:** long‐term survival, lung adenocarcinoma, next‐generation sequencing, pemetrexed, ROS1 rearrangement

## Abstract

Previously, cytotoxic drugs were the only option for patients with non‐small cell lung cancer (NSCLC) and the prognosis was poor. However, molecularly targeted therapies and immune checkpoint inhibitors represent a breakthrough in the treatment of advanced NSCLC and have improved survival rates. In addition, advances in next‐generation sequencing (NGS) have revealed the landscape of genomic alterations in patients with different cancers, aiding in the development of new molecularly targeted drugs. The patient reported here was a 54‐year‐old woman with left lower lung adenocarcinoma. The lung cancer was staged as T2aN3M1a stageIVA 11 years ago. She had received seven regimens of chemotherapy for 11 years. Among these, pemetrexed (PEM) regimens particularly showed long‐term effects totaling more than 5 years. We performed NGS after disease progression of the seventh treatment. NGS revealed CD74‐ROS1 fusion and she was treated with entrectinib. She has been taking entrectinib for over 20 months now. Herein, we report a rare case of CD74‐ROS1‐positive lung adenocarcinoma diagnosed by NGS that achieved long‐term survival with cytotoxic drugs, especially PEM regimens. In patients showing favorable clinical response to PEM regimens, physicians should consider testing for ROS1/ALK rearrangement.

## Graphical abstract

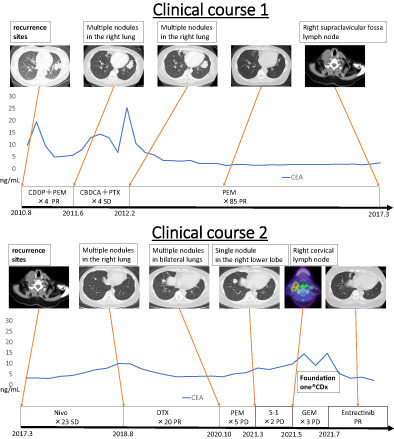

## INTRODUCTION

Lung cancer is the leading cause of mortality worldwide. The 1‐year survival rate after cytotoxic chemotherapy for patients with advanced non‐small cell lung cancer (NSCLC) is 29%,^1^ and long‐term (>10 years) survival is very rare. Lung cancer treatment has changed drastically in recent years owing to the advent of molecularly targeted therapies and immune checkpoint inhibitors (ICIs), and the survival rate of patients with lung cancer has significantly improved. Driver mutations are detected in more than 60% of NSCLC patients,^2^ and ROS proto‐oncogene 1 (ROS1) rearrangements are found in 1%–2% of patients with NSCLC. Furthermore, next‐generation sequencing (NGS) is now acknowledged worldwide, and new molecularly targeted drugs have been developed. Although, only 10%–20% of patients currently benefit from NGS,^3^ clinical benefit is expected to improve in the future. Herein, we report a case of CD74‐ROS1‐positive lung adenocarcinoma who achieved long‐term (>10 years) survival following cytotoxic drugs (especially PEM regimens) and ICIs and clinically benefited from NGS.

## CASE REPORT

**FIGURE 1:**
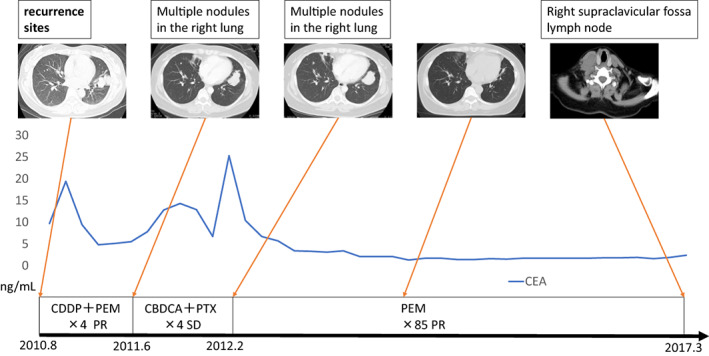
Patient's clinical course from 2010 to 2017. Radiological images (computed tomography and ^18^F‐fluorodeoxyglucose/positron emission tomography), serum carcinoembryonic antigen (CEA) level, treatment regimen, administration course and best response are shown.

**FIGURE 2:**
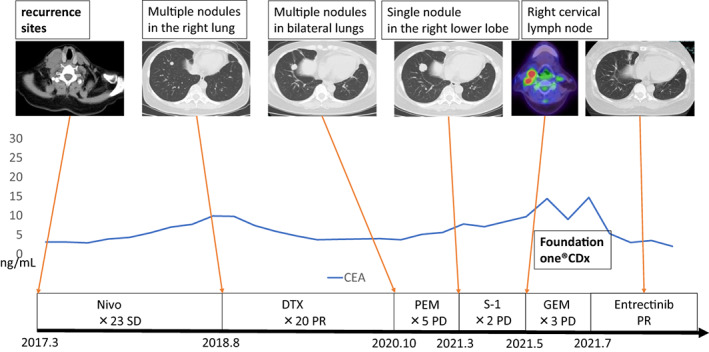
Patient's clinical course from 2017 to 2021. Radiological images (computed tomography and ^18^F‐fluorodeoxyglucose/positron emission tomography), serum carcinoembryonic antigen (CEA) level, treatment regimen, administration course and best response are shown.

**FIGURE 3:**
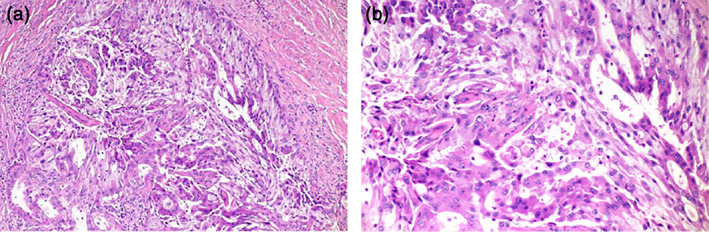
Images of hematoxylin and eosin‐stained tissues (magnification: A × 100, B × 200).

**FIGURE 4:**
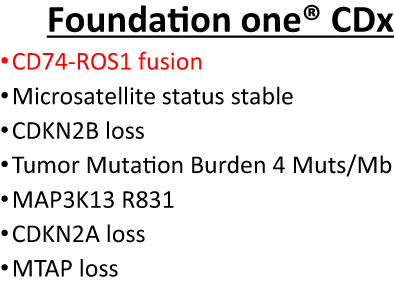
Foundation One CDx results.

A 54‐year‐old woman with left anterior chest pain had been diagnosed with left lower lung adenocarcinoma 11 years ago. She had a medical history of schizophrenia and 30 pack‐years of smoking history. The lung cancer was staged as T2aN3M1a (PUL) stageIVA. During the course of treatment, *EGFR* mutation was measured and was negative (cobas *EGFR* mutation test). She had received seven regimens of chemotherapy including cytotoxic drugs and ICI for 11 years (Figures 1 and 2). Among these, pemetrexed (PEM) regimens (cisplatine+PEM and PEM only) particularly showed long‐term effects, totaling more than 5 years. However, right cervical lymphadenopathy and right lower tumor enlargement were identified after two cycles of S‐1 as the seventh treatment line. We subsequently performed Foundation One CDx NGS using right cervical lymph node tissue (Figure 3), and gemcitabine (GEM) was selected as the eighth treatment line. However, right cervical lymphadenopathy and right lower tumor enlargement were observed after three cycles of GEM, and Foundation One CDx revealed CD74‐ROS1 fusion (Figure 4). Therefore, the patient was administered entrectinib as the ninth treatment line, which significantly reduced the right cervical lymphadenopathy and right lower tumors. She has been taking entrectinib for over 20 months now.

## DISCUSSION

ROS1 rearrangements in NSCLC were first detected in 2007 and are identified in 1%–2% of patients with NSCLC.^4^ Patients with ROS1 fusions tend be a middle‐aged, female, and never smokers or light smokers.^5^ There are more than 20 partner genes involved in ROS1 translocation, the most common of which is CD74.^6^ ROS1 and ALK are receptor tyrosine kinases and belong to the insulin receptor superfamily and each kinase domain has high homology.^7^ Therefore, ALK‐TKI is thought to be effective in NSCLC with ROS1 fusions. In the latest National Comprehensive Cancer Network (NCCN) guidelines, crizotinib, ceritinib and entrectinib are recommended as first‐line therapy in patients with ROS1‐positive metastatic NSCLC.^8^ However, in Japan, only crizotinib and entrectinib are approved for the treatment of patients with ROS1‐positive metastatic NSCLC.^9^ It has not yet been established which drug should be used first as a first‐line therapy. ROS1‐G2032R is the most frequently observed resistant mutation in NSCLC patients treated with crizotinib, showing a rate of 41%.^10^ ROS1‐G2032R is resistant to entrectinib both in vitro and in vivo,^11^ and subsequent treatment with entrectinib after disease progression on crizotinib is thought to be ineffective. Therefore, we administered entrectinib as the ninth treatment line ahead of crizotinib. The patient's subsequent clinical course has been good, with partial response (revised RECIST guidelines version 1.1) lasting more than 20 months.

Previous studies have reported that ALK‐positive patients had significantly longer progression‐free survival on PEM than ALK‐negative patients.^12^ Similarly, patients with ROS1 fusions showed better response to PEM than those without ROS1/ALK rearrangements.^13^, ^14^, ^15^, ^16^, ^17^ The thymidylate synthetase mRNA level was lower in patients with ROS‐1 positive than ROS‐1 negative patients, which explains the efficacy of PEM regimens.^15^ In our case, PEM regimens induced long‐term survival of over 5 years. In addition to this, treatment with nivolmab has been successful for 17 months. There are a few reports showing the efficacy of ICI for ROS‐1‐positive NSCLC.^18^, ^19^ The IMMUNOTARGET registry showed patients with ROS‐1 fusions have a high level of PD‐L1 expression.^18^ However, only limited number of cases have been reported and no confirmed evidence has been obtained. Further research is needed to clarify the role of ICIs in ROS‐1 positive NSCLC.

Over 60% of advanced NSCLC cases exhibit a targetable driver mutation,^2^ and young patients are more likely to harbor targetable genomic alterations.^20^ Furthermore, NGS development has enabled comprehensive, prompt, inexpensive genetic testing. Currently, only 10%–20% of patients with advanced cancer who undergo NGS testing receive sequencing‐directed therapy.^2^ However, new molecularly targeted drugs based on NGS testing are being increasingly developed, possibly improving clinical benefit in the future.

In this case, the remarkable efficacy of PEM regimens urged us to investigate other driver mutations, especially ROS1/ALK rearrangements.

In conclusion, we report a case of CD74‐ROS1‐positive lung adenocarcinoma diagnosed by NGS that achieved long‐term survival with PEM regimens. Patients with advanced NSCLC who achieve long‐term survival should be assessed for the presence of a driver mutation via NGS testing. In patients showing good clinical response to PEM regimens, physicians should consider testing for ROS1/ALK rearrangement.

## AUTHOR CONTRIBUTIONS

ST, NY, RA, ST, MY and KU drafted the manuscript and contributed to the treatment of the patient. All authors have read and approved the final manuscript.

## CONFLICT OF INTEREST STATEMENT

The authors declare no conflict of interest.

## PATIENT CONSENT

Written informed consent was obtained from the patient to publish this report in accordance with the journal's patient consent policy.
